# Combined genotypes of the *MBL2* gene related to low mannose-binding lectin levels are associated with vaso-occlusive events in children with sickle cell anemia

**DOI:** 10.1590/1678-4685-GMB-2016-0161

**Published:** 2017-08-21

**Authors:** Fernanda Silva Medeiros, Taciana Furtado de Mendonça, Katiuscia Araújo de Miranda Lopes, Laís Medeiros da Câmara França, Andreia Soares da Silva, Luydson Richardson Silva Vasconcelos, Maria do Carmo Valgueiro Costa de Oliveira, Ana Cláudia Mendonça dos Anjos, Betânia Lucena Domingues Hatzlhofer, Marcos André Cavalcanti Bezerra, Aderson da Silva Araújo, Patrícia Moura, Maria do Socorro de Mendonça Cavalcanti

**Affiliations:** 1 Instituto de Ciências Biológicas/Faculdade de Ciências Médicas, Universidade de Pernambuco, Recife, PE, Brazil; 2 Programa de Pós-graduação em Biotecnologia (RENORBIO), Universidade Federal Rural de Pernambuco, Recife, PE, Brazil; 3 Centro de Pesquisa Aggeu Magalhães (FIOCRUZ), Recife, PE, Brazil; 4 Hospital de Hematologia e Hemoterapia de Pernambuco (HEMOPE), Recife, PE, Brazil; 5 Universidade Federal de Pernambuco, Recife, PE, Brazil

**Keywords:** MBL2, polymorphism, sickle cell anemia, vaso-occlusive events

## Abstract

Sickle cell anemia (SCA) presents heterogenous clinical manifestations that cannot be explained solely by alterations to hemoglobin (Hb); other components such as endothelial adhesion, thrombosis and inflammation may be involved. The mannose-binding lectin (MBL) has an important role in innate immunity and inflammatory diseases. In this report, we describe an association between *MBL2* polymorphism related to low production of serum MBL and the frequency of vasoocclusive events (FVOE) in children ≤ 5 years old with SCA (p = 0.0229; OR 5.55; CI 1.11-27.66). Further studies are needed to explore the role of low MBL2 in the pathophysiology of vasoocclusive events in SCA.

In sickle cell anemia (SCA), the interaction between sickled erythrocytes and endothelial cells triggers inflammatory processes that lead to vaso-occlusive events (VOE) (Hebbel *et al.*, 2004). The mannose-binding lectin (MBL) is an important constituent of the innate immune system and is encoded by the *MBL2* gene. Variant alleles in the promoter (-550 H/L, −221 X/Y) and structural (minor alleles B, C and D) regions of *MBL2* influence protein expression and stability, respectively (Garred *et al.*, 2003). The structural exon-1 variant alleles B, C and D impair MBL oligomerization, leading to a reduction in binding capacity. Importantly, exon-1 codes for the collagen portion of the MBL molecule that binds cellular receptors and may influence phagocytosis and IL-10 production (Fraser *et al.*, 2006). MBL binds directly to cellular debris and pathogens and activates the complement system through mannan-binding lectin serine proteases (MASPs). This activation is an important step in the clearance of pathogens and/or injured cells and modulates the inflammatory response (Bohlson *et al.*, 2014).

Some studies have reported an association between *MBL2* polymorphisms and the clinical manifestations of chronic and degenerative diseases, including a positive association between *MBL2* variants and vaso-occlusive crises (VOC) in patients with SCA (Collard *et al.*, 2001; Oliveira *et al.*, 2009). In this study, we sought to confirm previous findings of our group (Oliveira *et al.*, 2009; Mendonça *et al.*, 2010) by evaluating the influence of *MBL2* polymorphism in structural and promoter regions over a broader range of clinical manifestations such as VOE in SCA patients.

This study was approved by the Research Ethics Committee for Studies in Human Beings of the HEMOPE Foundation (Registration no. 044/2008) and written informed consent was obtained from the patients. The study population consisted of 117 children with SCA, ranging from four months to five years old (median age: 3 years), with 50.74% being males. Clinical data were collected from the patients medical records at the HEMOPE Foundation and were recorded before initiating treatment with hydroxyurea (HU) and for assessment of the serum concentrations of MBL. Children who had received a transfusion in the previous three months were excluded.

Clinical events such as dactylitis, pain crises (episodes of pain), acute chest syndrome, acute splenic sequestration, stroke and priapism associated with SCA were designated VOE. The patients were classified in two groups based on the frequency of VOE (FVOE), as defined by Mendonça *et al.* (2010). This frequency was calculated as the ratio between the number of VOE and the age of the child at the end of the study, with FVOE ≥ 1 indicating severe disease.

Since a combination of *MBL2* genotypes in the −221 and exon 1 regions has previously been shown to be associated with the frequency of VOC (Mendonça *et al.*, 2010), in this investigation we measured serum MBL and investigated VOE as a phenotype. We also studied an additional polymorphism in the promoter region, the SNP −550 (H/L), which provided a more reliable indicator of the gene variability associated with serum levels (Garred *et al.*, 2003). The size of the study population in the present investigation was increased by 26% in relation to the number of patients studied by Mendonça *et al.* (2010).

DNA was extracted from peripheral blood, as previously described (Mendonça *et al.*, 2010). Exon 1 of *MBL2* was genotyped using real-time PCR with a melting temperature assay (MTA) (Mendonça *et al.*, 2010). The three allelic variants of the *MBL2* at codon positions 52, 54, and 57 in exon-1 were designated ‘O’ and the wild-type allele was designated ‘A’. The promoter region −221 (X/Y) rs7096206 and −550 (H/L) rs10031251 polymorphisms were detected using specific probes in conjunction with a TaqMan^®^ system (Applied Biosystems, Foster City, CA, USA). Real-time PCR was done using a Rotor Gene 6000^TM^ apparatus (Corbett Research Mortlake, Sydney, Australia). The primers, probes and validated protocols for these regions are available at http://snp500cancer.nci.nih.gov. The serum concentrations of MBL were determined using a commercial capture enzyme-linked immunosorbent assay (ELISA; Antibody Shop, Copenhagen, Denmark). The reactions were read at 450 nm using an ELISA plate reader (BioRad, Hercules, CA, USA). Based on the kit manufacturer's instructions, the concentrations of MBL in normal serum were classified as low (< 100 ng/mL), intermediate (100-1000 ng/mL) and high (> 1000 ng/mL) (Garred *et al.*, 2003). Since the patients with SCA were in Hardy-Weinberg equilibrium, the genotypes of the promoter region (-550 H/L, −221 X/Y) were grouped with those of exon 1 (A/O) and the correlation with MBL serum concentrations was used to classify the patients as having low, intermediate and normal MBL expression (Garred *et al.*, 2003).

There was a significant association between the genotypes and serum MBL concentrations (Figure 1) (Garred *et al.*, 2003). The genotype combinations related to low MBL levels were associated with an FVOE ≥ 1 (p = 0.0229; OR = 5.55; 95%CI = 1.11-27.66) (Table 1). The frequencies of the genotypes (-550 HL and H allele) associated with high serum MBL concentrations were higher in the group with a lower frequency of VOE (p = 0.0129; OR = 2.38; 95%CI = 1.15-5.15 and p = 0.0292; OR = 1.76; 95%CI = 0.97-3.19, respectively). Likewise, in the −221 region the frequency of the YX genotype related to low serum MBL levels was higher in patients with FVOE ≥ 1 compared to FVOE < 1 (37% *vs.* 21%), thus confirming previous findings by our group. Oliveira *et al.* (2009), in an analysis of the relationship between the genotypes of *MBL2* exon 1 in children with SCA identified an association between the AO/OO genotypes and VOC (p = 0.039; OR = 3.01; 95%CI = 1.05-9.11). In addition, this population showed an association between polymorphism in the promoter region −221 and exon 1 of *MBL2* in relation to VOC (p = 0.0188; OR = 3.15; 95%CI = 1.19-8.50) (Mendonça *et al.*, 2010).

**Figure 1:**
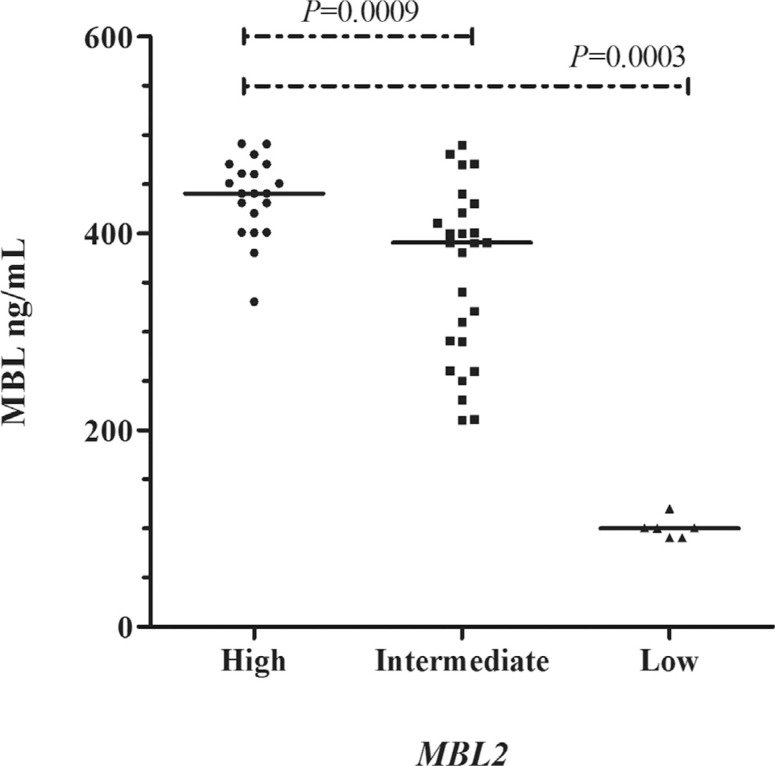
Levels of serum mannose-binding lectin (MBL) in patients with sickle cell anemia according to the genotypes of the promoter and exon-1 of the *MBL2* gene. Mann-Whitney test. A: wild-type allele, B, C and D: variant alleles commonly designated as allele O; L and Y: wild-type alleles; H and X: variant alleles. The following combined *MBL2* genotypes were considered to yield high MBL concentrations: HYA/HYA, HYA/LYA and LYA/LYA (median = 440.4, range = 330.5-490.9, n = 20), intermediate concentrations: HYA/HYO, HYA/LXA, HYA/LYO, LYA/LXA, LXA/LXA, LYA/LYO and HYO/LYA (median = 390.4, range = 210.3-490.0, n = 26) and low concentrations: HYO/LXA, LXA/LYO and LYO/LYO (median = 100.3; range = 90.6-120.3, n = 6).

**Table 1:** Association of combined genotypes of *MBL2* in relation to the serum levels of MBL and the frequency of vaso-occlusive events in children with sickle cell anemia.

*MBL2*	SCA N = 126 (%)	FVOE ≥ 1 N = 68 (%)	FVOE < 1 N = 58 (%)	*p*	OR	95%CI
Promoter SNP −550						
LL	76 (0.60)	47 (0.69)	29 (0.50)	Reference		
HL	42 (0.33)	17 (0.25)	25 (0.43)	0.0129*	**2.38**	**1.10–5.15**
HH	08 (0.07)	04 (0.06)	04 (0.07)	0.3856	1.62	0.37-6.98
L	194 (0.77)	111 (0.82)	83 (0.72)	Reference		
H	58 (0.33)	25 (0.18)	33 (0.28)	**0.0292** *	**1.76**	**0.97-3.19**
Promoter SNP −221						
YY	86 (0.68)	42 (0.62)	44 (0.76)	Reference		
YX	37 (0.29)	25 (0.37)	12 (0.21)	0.0279*	**0.46**	**0.20-1.03**
XX	03 (0.03)	01 (0.01)	02 (0.03)	0.5256	1.90	0.16-21.86
Y	209 (0.83)	109 (0.80)	100 (0.86)	Reference		
X	43 (0.17)	27 (0.20)	16 (0.14)	0.1012	0.64	0.32-1.13
Exon 1						
AA	87 (0.69)	44 (0.65)	43 (0.74)	Reference		
AO	37 (0.29)	23 (0.34)	14 (0.24)	0.1181	0.62	0.28-1.36
OO	02 (0.02)	01 (0.01)	01 (0.02)	0.7472	1.02	0.06-16.90
A	211 (0.84)	111 (0.82)	100 (0.86)	Reference		
O	41 (0.16)	25 (0.18)	16 (0.14)	0.1626	0.71	0.36-1.14
Haplotypes of MBL2						
High (HYA + LYA)	168 (0.67)	84 (0.62)	84 (0.72)	Reference		
Intermediate (LXA)	43 (0.17)	27 (0.20)	16 (0.14)	0.0670	0.59	0.29-1.18
Low (HYO + LYO)	41 (0.16)	25 (0.18)	16 (0.14)	0.1036	1.56	0.77-3.13
Combined genotypes (diplotypes) of MBL2						
High	57 (0.45)	27 (0.40)	30 (0.52)	Reference		
Intermediate	57 (0.45)	31 (0.46)	26 (0.45)	0.2268	1.31	0.61–2.82
Low	12 (0.10)	10 (0.14)	02 (0.03)	**0.0229** **	**5.55**	**1.11–27.66**

SCA - Sickle cell anemia; FVOE - Frequency of vaso-occlusive events (VOE), defined as the ratio of total episodes divided by the age of the children at the end of this study, adapted according to Mendonça *et al.* (2010). Patients with < 1 event per year were defined as FVOE < 1/year (mild disease), those with ≥ 1 events per year were defined as FVOE ≥ 1/year (severe group). Combined genotypes of *MBL2*: high (HYA/HYA, HYA/LYA, LYA/LYA), intermediate (HYA/HYO, HYA/LXA, HYA/LYO, LYA/LXA, LXA/LXA, LYA/LYO, HYO/LYA) and low (HYO/LXA, LXA/LYO, LYO/LYO) MBL levels. A – wild allele, O – variant allele, L and Y – wild-type alleles, H and X – variant alleles, SNP – single nucleotide polymorphism. Significant results are shown in bold, the

* Chi-square or

** Fisher's exact test were used.

The association of low serum MBL with increased FVOE in children with SCA may be explained by the ability of MBL to activate the complement system independently of antibody production. This activation can regulate the inflammatory response in the endothelium, particularly since MBL stimulates the removal of sickled cells, thereby controlling chronic inflammation (Platt, 2000; Nauta *et al.*, 2004). In activating the complement system to remove sickled cells, MBL induces IL-10 production, thereby avoiding unnecessary stimulation of the immune system. MBL can also diminish the exposure of surface autologous IgG bound to denaturated Hb (Hebbel *et al.*, 1980; Kannan *et al.*, 1988) and consequently attenuate the erythrophagocytosis of sickle cells (Hebbel and Miller, 1984).

Our results generally support the idea that MBL plays an important role early in life that may be related to microorganisms and the clearance of immune complexes and sickled erythrocytes. This function may be supplemented over time by maturation of the adaptive immune system (Tsutsumi *et al.*, 2005). In fact, Zachariah *et al.* (2016) found an association between *MBL2* polymorphism and infection in patients with sickle cell disease, although there was no association with VOC. This may reflect differences in the patients' characteristics, particularly their age and the criteria for defining cases.

SCA patients show a high consumption of serum complement components that is partly related to the opsonization of sickled cells (Wilson *et al.*, 1979). Patients deficient in MBL are more likely to have defective phagocytosis and this can lead to the accumulation of sickled erythrocytes in the vessel wall, thereby contributing to the occurrence of VOE. Over the years, other components of the adaptive immune system, such as the complement classical pathway, may compensate for this deficient clearance of sickled cells (Bensinger and Gillette, 1974).

Interestingly, a study of *MBL2* variants in Benin, West Africa, found a higher frequency of heterozygosis, mainly in the allele C, in adults compared to newborns with SCA (47% *vs.* 35.3%; p = 0.004) (Dossou-Yovo *et al.*, 2007). This finding may be indicative of a selective advantage for this variant in African patients. In this scenario, intermediate levels related to heterozygosis should balance the role of MBL in modulating the inflammatory response. Indeed, as indicated here, the HL genotype showed a higher frequency in patients with a better outcome, thus supporting this hypothesis.

A deficiency in MBL may cause greater systemic injury by increasing the frequency of VOE episodes during early childhood and predisposing the individual to severe complications. Our results suggest that combined genotypes associated with low MBL serum levels may be useful markers for severity and could contribute to our understanding of the clinical diversity of SCA, in addition to stimulating studies with MBL as a possible immunotherapeutic molecule.
